# Hardness and Elastic Modulus on Six-Fold Symmetry Gold Nanoparticles

**DOI:** 10.3390/ma6010198

**Published:** 2013-01-14

**Authors:** Manuel Ramos, Luis Ortiz-Jordan, Abel Hurtado-Macias, Sergio Flores, José T. Elizalde-Galindo, Carmen Rocha, Brenda Torres, Maryam Zarei-Chaleshtori, Russell R. Chianelli

**Affiliations:** 1 Departmento de Física y Matemáticas, Instituto de Ingenieríay Tecnología, UACJ, Avenida del Charro #450 Nte. Cd. Juárez, Chihuahua, C.P. 32312, Mexico; E-Mails: seflores@uacj.mx (S.F.); jose.elizalde@uacj.mx (J.T.E.-G.); 2 Materials Research and Technology Institute, University of Texas at El Paso, 500 W. University Ave, El Paso TX 79968, USA; E-Mails: lortiz161@email.suagm.edu (L.O.-J.); crrocha2@miners.utep.edu (C.R.); btorres2@utep.edu (B.T.); mzarei@utep.edu (M.Z.-C.); chianell@utep.edu (R.R.C.); 3 Centro de Investigación en Materiales Avanzados S.C., Laboratorio Nacional de Nanotecnología-Chihuahua Miguel de Cervantes 120, Complejo Industrial Chihuahua, Chihuahua, Apdo. Postal 31109, Mexico; E-Mail: abel.hurtado@cimav.edu.mx

**Keywords:** nanoparticles, gold, HRTEM, nanoindentation, hardness

## Abstract

The chemical synthesis of gold nanoparticles (NP) by using gold (III) chloride trihydrate (HAuCl∙3H_2_O) and sodium citrate as a reducing agent in aqueous conditions at 100 °C is presented here. Gold nanoparticles areformed by a galvanic replacement mechanism as described by Lee and Messiel. Morphology of gold-NP was analyzed by way of high-resolution transmission electron microscopy; results indicate a six-fold icosahedral symmetry with an average size distribution of 22 nm. In order to understand the mechanical behaviors, like hardness and elastic moduli, gold-NP were subjected to nanoindentation measurements—obtaining a hardness value of 1.72 GPa and elastic modulus of 100 GPa in a 3–5 nm of displacement at the nanoparticle’s surface.

## 1. Introduction 

The synthesis and fabrication of metallic nanoparticles has been approached with high success by using wet-chemistry methods when targeting specific applications (*i.e.*, Catalysis, Cancer, and Optics) [1,2,3]. Reactions like the galvanic replacement, as proposed by Lee and Meisel, seem to be ideal in achieving large frequency spherical-like nanoparticles, using metallic salt precursors [4]. Three basic shapes are formed on monometallic nanoparticles: decahedral, cubo-octahedral, and icosahedral. Their final morphology has to do with the lowest surface energy *γ* in the (111)-plane, however it implies that large internal core-strain values are necessary; leading to the conclusion that nanoparticle surface are constituted by *γ*(111) *<γ*(100) *<γ*(110) as indicated by Elechiguerra *et al.* [5]. 

Nanoparticles geometry and facets are made out of (111) planes as observed in icosahedron; and is attributed to lowest surface energy *γ*(111) of nucleation in (111)- plane. This implies large facets, whereas decahedron has moderate internal strain and smaller facets made of (111) and (100) planes [6]. Theoretical investigations for heat of formation, reactivity, kinetics and mechanical behavior are possible to calculate using molecular dynamic computational methods. Recently, Casillas *et al.* were able to do a dynamic simulation on gold twinned nanoparticles with five-fold symmetry, by applying dynamic stress at different directions and locations. This indicates the formation of dislocations and stacking faults in the range of 0 to 4 GPa of external stress applied [7]. 

Carlton and Ferreira presented a similar study using *in situ* HRTEM technique [8] by deforming a silver nanoparticle with the aid of a sample holder equipped with a nanoindenter manipulator. HRTEM images reflect a dislocation type a/2 {1 1 0} on {1 1 1} type plane of nanoparticle, where a correlation between high resolution images and mechanical deformation is present. Indications of staking faults and dislocations were also observed in their study. In similar fashion, a study to determine elastic moduli in spherical silicon nanoparticles—using the nanoindentation technique—was presented by Mook *et al.* [9]. In their study it was assumed that there was uniform and rigid contact between nanoindenter tip and substrate, thus contribution of stress-strain comes from silicon nanoparticle only and the elastic modulus was calculated using average true-strain/true-stress relationships methods [10]. Here, the usage of nanoindentation technique through Continuous Stiffness Measurement (CSM) method as described by Li *et al.* [11] is used to measure mechanical properties on gold nanoparticles (Au-NP) with characteristics of six-fold symmetry as revealed by HRTEM images. 

## 2. Results and Discussion 

### 2.1. High Resolution Transmission Electron Microscopy

**Figure 1:**
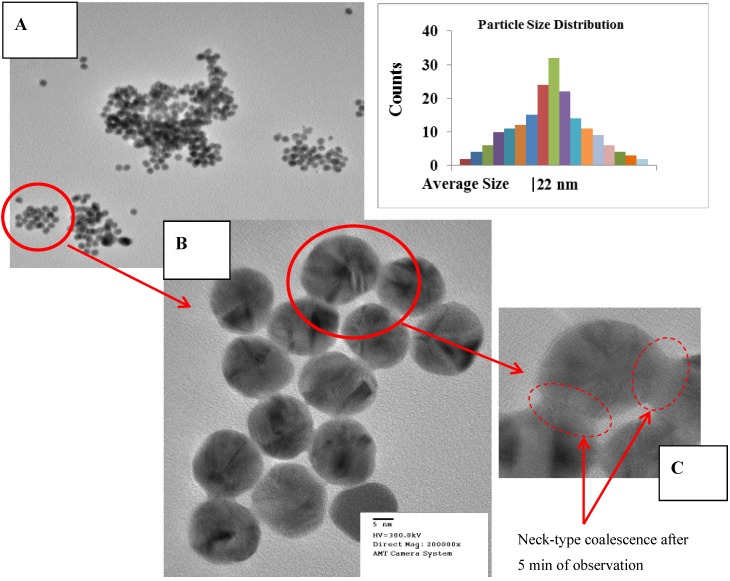
(**a**) HRTEM image indicating the agglomeration of Au-NP. (**b**) 5 nm of resolution HRTEM image on Au-NP six-fold symmetry is observed. (**c**) Coalescence effect start to occurring after 2 min of observation, due to high electron beam dosage, typical formation of neck-type between walls of neighbor Au-NP’s.

Morphology, average size and symmetry of gold nanoparticles (Au-NP) were investigated by field emission gun electron microscopy techniques. Images were taken at 300 kV with a current set to 10 μA. The equipment used was a Hitachi H-9500 equipped with EDX, X-twin lenses and CCD camera. Interpretation of the images and measurements was done using Digital Micro computational package. Results indicate average size diameter of 22 nm with a six-fold symmetry, as presented in Figure 1a,b. It was understood during HRTEM measurements that Au-NP morphological surface is very sensitive, thermodynamically speaking, to electron gun dosage since it creates some coalescence effect between them, as shown in Figure 1b. Yacaman *et al.* has reported this effect at temperatures ranging from 283 K to 830 K by applying energetic effects from electron beam due to the interactions with nanoparticles and electrons (phonons) [12]. 

### 2.2. X-ray Diffraction and Infrared Absorption 

**Figure 2:**
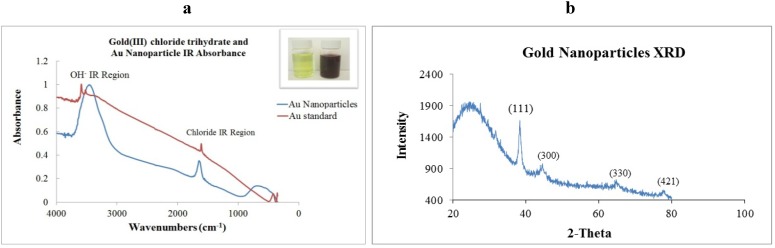
(**a**) Fourier Transform Infrared spectra of colloidal gold (red) and Au-NP (blue) Inset: Photographic image indicating change in coloration. (**b**) X-ray diffraction peaks indicating principal reflections at (111), (300), (330), (421).

For further characterization purposes, Au-NP was subjected to both measurement techniques. Powder X-ray diffraction was performed using a Panalytical XPert PRO machine, Cu_-Kα_ radiation, step size of 0.016° and step time of 30 s. Infrared absorption was done using a benchtop Thermo Nicolet model nexus 470-FTIR and OMNIC package. In Figure 2a, a comparison of IR spectra is presented for gold (III) chloride trihydrate before reaction and Au-NP formation. Two main broad peaks are found at 3450 and 1650 cm^−1^. For XRD principal reflections are (111), (300), (330) and (421). Broader shoulder was found at *2θ* = 25°—corresponding to liquidity of the sample and texture created by Au-NP when deposited onto holder as presented in Figure 2b. 

### 2.3. Mechanical Nanoindentation

Mechanical nanoindentation as a method to study mechanic-structural properties in nanoparticles has brought so much attention in the past five years [13,14]. Investigations of nano-regime for specific nanostructures (*i.e.*, nanoparticles, nanowires, nanorods) and their comparison with bulk materials seem to be the objective when understanding structure/function relationships and mechanical behavior. 

**Figure 3:**
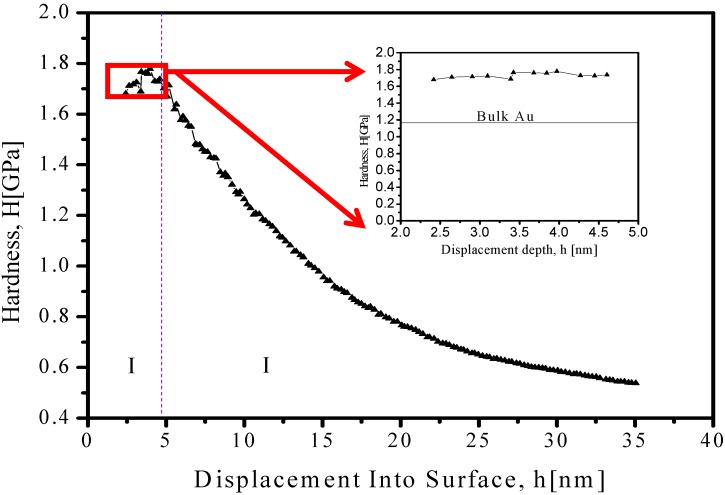
Measurements of hardness and function of displacement from CSM nanoindentation of Au-NP. Inset: Specific region between 2.5–6.0 nm of displacement, indicating a 1.72 ± 0.03 GPa value. Horizontal dotted line corresponds to values found for gold in bulk phase [18].

The Young modulus and hardness of the Au-NP were obtained using Agilent Nano Indenter (inset in Figure 3) G200—equipped with a standard configuration and DCM indentation head. All measurements were performed using Continuous Stiffness Measurement (CSM) method as described by Li *et al.* [15].

Equipment was calibrated using a standard fused silica with the following test parameters: Berkovich diamond indenter with tip radius of 20 nm, deep limit of 35 nm, strain rate target of 0.05 1/s, harmonic displacement target of 1 nm and a frequency target of 75 Hz. 

Mechanical behavior of Au-NP was subjected to study using nanoindentation as described above in order to measure hardness (H), elastic modulus (E), and elastic stiffness (S). Traditionally, S could be determined from the slope of the load- displacement data acquired during unload [16]. However, using this approach, one can obtain the S values and H, E can only be determined when maximum penetration depth is reached, thus CSM option enables a continuous measure of S during loading—and not just at the point of initial unloads [17]. The latter is accomplished by superimposing a small oscillation on the primary loading signal and analyzing the resulting response of the system by means of a frequency-specific amplifier. Equation 1 presents the mathematical model used to determine CSM.

(1) $$S={\left|\frac{1}{\frac{F_{0}}{Z_{0}}\text{cos}\emptyset -\left(K_{S}-m\omega^{2}\right)}-\frac{1}{K_{f}}\right|}^{-1}\;$$

*ω* represents the excitation frequency. If the displacement amplitude (*Zo*), phase angle (∅), and excitation amplitude (*Fo*) is measured and determined machine parameters, such as load-frame stiffness *K_f_*, support springs stiffness *Ks*, and mass m (See inset of Figure 3a)—the hardness and elastic modulus as a continuous function of surface penetration can be obtained. Therefore, CSM option is useful to evaluate mechanical behavior on thin films, as well for this case when gold nanoparticles are mounting over silicon substrates. Samples for nanoindentation measurements were prepared by depositing one drop of colloidal solution over clean silicon wafer area, letting dry at room temperature under glove box conditions in order to avoid contaminants. The data from loads is presented in Figure 3. Inset presents an enlargement of 2.5 nm to 6 nm displacement on penetration depth, where a hardness value of 1.72 ± 0.03 GPa is obtained, which is close to bulk phase found experimentally to be 1.11 ± 0.03 GPa as presented by Volinsky *et al.* [18]. 

**Figure 4:**
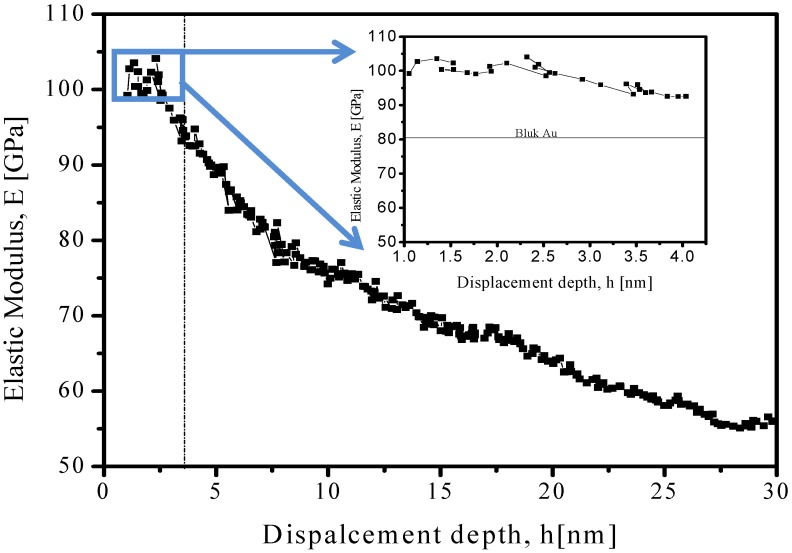
Measurements of elastic modulus as function of displacement from CSM nanoindentation of Au-NP. Inset: Specific region between 1.0–4.0 nm of displacement, indicating a 100 ± 2 GPa value. Horizontal dotted line corresponds to values found for gold in bulk phase [18].

The contribution for elastic modulus is presented in Figure 4, with a specific average value 100 ± 2 GPa—higher in comparison to bulk materials as well presented by Volinsky *et al.* [18]. 

Unfortunately, one of the nanoindenter equipment limitations is the capacity to do *in situ* microstructural observations during the mechanical testing. In order to record direction of dislocation, as presented by others Casillas [7], Ferreira [8] and Deneen [15], when using electron microscopy techniques, no further discussion can be made about dislocations, stacking faults, bulk phase change and twin boundaries on this systems of nanoparticles. 

## 3. Experimental Section 

Gold nanoparticles were synthesized by using a mixture of gold (III) chloride trihydrate (HAuCl_4_·3H_2_O), sodium hydroxide (NaOH), sodium citrate (C_6_H_5_Na_3_O_7_) and distilled water. A solution of 2.5 mM of HAuCl_4_·3H_2_O was prepared by mixing 0.05 g of gold (III) chloride trihydrate in 50 mL of deionized water. A second solution of 6.6 Mm sodium hydroxide (NaOH) was prepared by mixing 0.0264 g of NaOH in 100mL of deionized water. The pH of the 10 mL gold solution was controlled by adding NaOH until the pH reached 6.6. The solution was heated to 85 °C under vigorous stirring for 30 min. Then a solution of 5 mM of sodium citrate (C_6_H_5_Na_3_O_7_) was prepared by mixing 0.073 g of sodium citrate with 50 mL of deionized water and heating 10 mL solution of sodium citrate to around 85 °C before adding it to the gold solution under vigorous mixing. The reaction was maintained at 85 °C for approximately 20 min. This step reduced the gold (Au^+3^→Au°) to produce the nanoparticles and the solution turned ruby red. The galvanic reaction is produced in agreement to:

(2) $$HAuCl_{4}\cdot 3H_{2}O+C_{6}H_{5}Na_{3}O_{7}+NaOH\to Au^{0}+4NaCl+C_{6}H_{5}Na_{2}O_{7}+4H_{2}O$$

## 4. Conclusions 

Mechanical properties of Au-NP are presented here by means of nanoindentation with the continuous stiffness method. Morphological aspects of gold nanoparticles were studied by way of HRTEM techniques, indicating a six-fold symmetry nanoparticle type, with average size of 22 nm. Using the nanoindentation technique it was possible to measure the Elastic modulus and hardness values which are found to be ***E*** = 100 ± 2 GPa with an standard deviation of 3.6353 and ***H*** = 1.72 ± 0.03 GPa with standard deviation of 0.0306 respectively, indicating higher mechanical strength when compared to bulk phase. Unfortunately, the nanoindenter equipment used to perform measurements is not equipped with an imaging interface, which is needed to detect any microstructural change due to external forces. Therefore, a detailed understanding of atomistic behavior is not presented here, as it is presented in literature [8,15,19]. A trivial conclusion could be the formation of stacking faults and dislocations preferentially on the <111> direction of nanoparticles. For this reason, further work is under progress by using molecular dynamics computational methods to simulate mechanical testing and comparison of theoretical and experimental data.
